# Three-Dimensional *Ex Vivo* Culture for Drug Responses of Patient-Derived Gastric Cancer Tissue

**DOI:** 10.3389/fonc.2020.614096

**Published:** 2021-02-15

**Authors:** Sian Chen, Chenbin Chen, Yuanbo Hu, Ce Zhu, Xiaozhi Luo, Lizhu Wang, Xiang Wang, Xiangwei Sun, Xiaodong Chen, Wangkai Xie, Han Lou, Xielin Huang, Chao Li, Jun Xu, Xiangyang Xue, Xian Shen

**Affiliations:** ^1^ Department of Gastrointestinal Surgery, The Second Affiliated Hospital & Yuying Children’s Hospital of Wenzhou Medical University, Wenzhou, China; ^2^ Department of Microbiology and Immunology, School of Basic Medical Sciences, Institute of Molecular Virology and Immunology, Wenzhou Medical University, Wenzhou, China; ^3^ Department of Pathology, The Second Affiliated Hospital & Yuying Children’s, Hospital of Wenzhou Medical University, Wenzhou, China; ^4^ The First School of Medicine, Wenzhou Medical University, Wenzhou, China

**Keywords:** gastric cancer, *ex vivo* tumor tissue culture, proliferation, apoptosis, chemotherapy effectiveness

## Abstract

Gastric cancer (GC) is one of the most common malignancies with high mortality and substantial morbidity. Although the traditional treatment strategies for GC revolve around surgery, radiotherapy, and chemotherapy, none have been able to optimally treat most affected patients. To improve clinical outcomes and overcome potential GC resistance, we established a three-dimensional (3D) culturing platform that accurately predicts drug responses in a time- and cost-effective manner. We collected tumor tissues from patients following surgeries and cultured them for 3 days using our protocol. We first evaluated cell proliferation, viability, and apoptosis using the following markers: Ki67 and cleaved caspase 3 (Cas3). We demonstrated that cell viability was maintained for 72 h in culture and that the tumor microenvironments and vascular integrities of the tissues were intact throughout the culture period. We then administered chemotherapeutics to assess drug responses and found differential sensitivity across different patient-derived tissues, enabling us to determine individualized medication plans. Overall, our study validated this rapid, cost-effective, scalable, and reproducible protocol for GC tissue culture that can be employed for drug response assessments. Our 3D culture platform paves a new way for personalized medication in GC and other tumors and can greatly impact future oncological research.

## Introduction

GLOBOCAN reported that, in 2018, there were approximately 1,033,000 new cases of gastric cancer (GC) (1/18 of all cancers) and 783,000 GC-related deaths (1/12 of all cancer-related death) worldwide. GC ranks 5th in the incidence of malignant tumors and 2nd in mortality (1). The 2015 China Cancer Data Report stated that there were 679,000 new cases of GC and 498,000 GC-related deaths in China, accounting for over 50% of the GC morbidity and mortality in the world. In China, from 2011 to 2015, the incidence of GC increased by 30.1 and 21.7% in males and females, respectively, and exhibited an upward tendency (2). China has become the country with the highest risk for GC in Asia (3).

Depending on the clinical stage, traditional therapeutic strategies such as surgery, radiotherapy, and chemotherapy do not completely eradicate GC lesions. Metastasis, recurrence, and subsequent chemoresistance are still the major causes for GC-related fatalities (4). To improve the 5-year survival rate, after surgery, patients undergo combined chemotherapies under the guidelines of international classic chemotherapy or empirical strategies (5). Even so, only 5% of patients have benefitted with an increased 5-year survival rate, and GC prognosis continues to be poor (5, 6). Therefore, there is urgency to develop rapid and accurate strategies to predict the chemotherapy effects of individual medication and improve clinical outcomes.

Immortalized cancer cell lines (such as Hela cells) and *in vitro* 2-dimensional culture (2D) techniques are widely used in oncology research, especially to study pharmacokinetics; these cancer cell lines have played important roles in drug sensitivity, *in vivo* efficacy prediction, and prognosis evaluation (7). *In vitro* 2D preclinical tumor models have helped decipher the reasons for malignant transformations and the emergence of chemoresistance, greatly impacting clinically translatable findings. However, these *in vitro* studies are not directly translatable, often leading to poor clinical outcomes or are rendered completely ineffective in patients, demonstrating a clear disconnect between preclinical and clinical models (8, 9).

Additionally, cancer cells grow in a comprehensive 3-dimensional (3D) matrix and cope with a series of biophysical and biochemical factors that naturally coexist in this complex milieu, such as bioadhesivity, stiffness, extracellular matrix (ECM), and adhesion molecules (integrins) (10–12). The use of traditional 3D culture systems such as the rotary cell culture system, floating cell culture, and hydrogel scaffold culture system is limited. This is because replicating the spatiotemporal niches, the inherent heterogeneity in cancer cells, and the surrounding ECM remains challenging (13, 14). Hence, the development of 3D *ex vivo* culture protocols, based on patient-derived tumor tissues, is necessary to properly assess tissue and cell viability, and optimize timelines in pharmacological research (15).

Patient-derived tumor xenograft (PDX) models have been used as alternative preclinical models, as they closely resemble tumor phenotypes and heterogeneity. They possess clear advantages over traditional models and are effective for developing medical strategies and predicting clinical prognoses (16). PDX models have played essential roles in personalized drug development and accurate susceptibility studies (17). However, not all cells can be proliferated in PDX models, such as different subsets of tumor and stromal cells, due to the natural heterogeneity of the native tumor tissues. Only a few unique subsets of tumor cells that are adaptable to the xenograft host environment can be developed into PDX models (18). Therefore, PDX models only partially reflect the cellular characteristics of native tumor tissues and are not complete representatives of heterogeneity within the tumor milieu (19). Additionally, the establishment of PDX models is time-consuming (at least 3 months), has a low success rate (~30%), is expensive, and is not amenable for high throughput patient screening (20).

Although molecular profiling has identified diverse signaling pathways in GC subtypes, clinical treatments still largely depend on standard regimens (21). Accurate and precise medications have been increasingly matched against biogenetic characteristics of individual patients (22, 23). However, no current protocol predicts the patients’ responses to primary chemotherapeutics for GC. For primary and recurrent tumors, clinical treatments are still based on standard regimens, including first- and second-line chemotherapies (24). Therefore, there is an increasing need for more accurate and precise preclinical models to predict individualized therapeutic responses.

In a previous study by Koerfer et al. (25), a culture model of human gastric and esophagogastric junction cancer using a tissue chopper was established. However, a tissue chopper is not common in most hospitals, and this process is time-consuming and depends on potentially error-prone tissue sectioning. Therefore, we investigated a novel and convenient culture system that did not require the use of a tissue chopper. Our 3D culture system retained the intact tumor microenvironment, reflected real tumor heterogeneity, was comparable to native tumor tissues, and maintained cell viability similar to the *in vivo* status for at least 72 h.

## Materials and Methods

### Patients and Tumor Tissue Collection

The study was approved by the ethics committee of the Second Affiliated Hospital of Wenzhou Medical University. Tissue was collected from 25 patients with GC during their surgeries. The characteristics of the 30 cases of GC are listed in **Table S1**. The GC samples were cultured and further examined on a daily basis. Normal regions near the cancer were rejected by the surgeon and confirmed by a board-certified pathologist. Tissue pieces were collected and immersed in 20 ml phosphate buffered saline (PBS, Gibco, Grand Island, NY, USA, 10010049) containing 1% penicillin/streptomycin (100×, Gibco, Grand Island, NY, USA, 10378016), and transported to the laboratory within 10 min for further processing.

### Tissue Preparation and Culture

Continuous and accurate collection of the patient-derived tumor tissues is the decisive factor for reliable parameter comparison in the culture process (26). To ensure continuity of the GC tissue, each sample was accurately trimmed to remove the necrotic tissue and unrelated mucosa (27), which are unsuitable for culturing.

**Figure 1:**
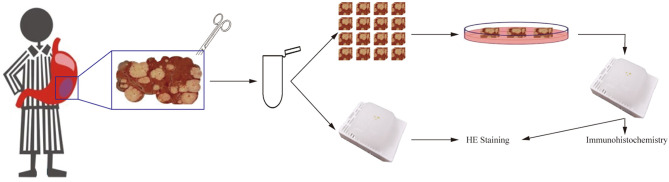
Tumor tissue collection, preparation, and culture.

For preparation, tissues were washed twice with PBS to remove the blood and necrotic surface residues. They were then cut into pieces in a 4 ml tube using ophthalmic scissors, on ice. Tissues (0.8–1.2 mm in diameter) were picked up under a binocular stereo microscope and transferred into six-well plates (four to six tissues per well and one well per group). Each well contained 1 ml of culture medium, with the lower parts of the tissues submerged and the upper parts exposed to air. No scaffold was used to support the tissue pieces in the wells (**Figure 1**). The complete medium was supplemented with RPMI-1640 (Gibco, Grand Island, NY, USA, 31870082), 10% fetal calf serum (Gibco, Grand Island, NY, USA, 10099141C), 50× B-27™ (Gibco, Grand Island, NY, USA, 17504044), 1% l-glutamine (Gibco, Grand Island, NY, USA, 25030081), and 1% penicillin/streptomycin (Gibco, Grand Island, NY, USA, 10378016). Tissues were incubated in a humidified incubator at 37°C and 5% CO2 for 1 to 3 days. The culture medium was changed daily. Tissues fixed on the preparation day were labeled as Day 0 of isolation.

### Drug Responses

We used Oxaliplatin (10.0 μg/ml, MedChemExpress, Monmouth Junction, USA, HY-17371) and 5-Fluorouracil (5-FU, 10.0 μg/ml, Aladdin Shanghai, China, F100149) to test drug responses. The medium was changed every day and the drug concentration was maintained consistently. After 3 days of culture, tissues were fixed, paraffin-embedded, and sectioned (5 μm). Hematoxylin and Eosin (H&E) staining and immunohistochemical staining were carried out as described below. The cell proliferation and apoptotic indices of tissues cultured with chemotherapeutics were quantitatively evaluated and compared with the indices of tissues cultured in parallel without exposure to chemotherapeutics (control).

### Tissue Fixation and H&E Staining

On the day of surgery and on days 1–3 of the culture process, small pieces of tissue were taken, fixed in 4% paraformaldehyde for 24 h, and labeled as Day 0, 1, 2, and 3 of isolation, respectively. Fixed tissues were embedded in paraffin, sectioned, and stained with an H&E kit (Scientific Phygene, Fuzhou, China, PH0516). The tissue structural and morphological changes were compared and recorded daily.

### Immunohistochemistry Staining

Cell type and distribution were determined by H&E staining and immunohistochemical staining. The specific experimental steps refer to the previous literature of our team (28). Primary and secondary antibodies are listed in **Table S2**. For antigen retrieval, the paraffin tissue sections were immersed in citrate buffer (pH 6.0), and placed in a pressure cooker until steam was generated for 3 min. To block non-specific peroxidase, a 3% hydrogen peroxide and methanol solution was applied to tissue sections for 10 min, following incubation in goat serum (Solarbio Life Science, Beijing, China, S9070) for 30 min. Tissue sections were then incubated with diluted primary antibody (**Table S2**) at 4°C overnight and washed twice with PBS. Fluorescent dye-labeled or HRP-labeled secondary antibodies were applied to the tissue sections for 30 min, followed by three PBS washes. The nuclei were counterstained with 2-(4-amidinophenyl)-6-indolecarbamidine dihydrochloride (DAPI) (Solarbio Life Science, Beijing, China, C0065) or DAB (Dako, Glostrup, Denmark, 20052898) and hematoxylin (Scientific Phygene, Fuzhou, China, PH1464). Images of all sections were captured using a Leica DM2500 microscope.

### 5-Ethynyl-2’-deoxyuridine (EdU) Incorporation

Tissues were collected and cultured in medium containing 50 μM EdU for 2 h at 37°C before fixation. Detection of incorporated EdU was performed with the Cell-Light EdU Apollo 488 kit (RiboBio, Guangzhou, China, C10310-3), according to the manufacturer’s protocol.

### Analysis and Statistics

After H&E staining, the histopathological classification and cell type were confirmed by a board-certified pathologist. During the culture process, to evaluate cell viability in tissue, H&E staining was performed to determine the total cell counts every day. Cas3 was used as an immunohistochemical marker of apoptosis and Ki67 was used as an immunohistochemical marker of proliferation. The tumor cell fraction tissue integrity was determined with Keratin20 and CD133 staining. The positive cells were counted using Image Pro Plus software (Media Cybernetics, v6.0), and reconfirmed by manual counting, and the positive area was also calculated with the Image Pro Plus software. Due to the difference in proteins expression position, CD133, p53, HIF-1α, EdU, and Ki67 positive rates were calculated as follows: positive cell number of immunohistochemistry/total cell number of immunohistochemistry. Keratin20, Fibronectin, Collagen1, α-SMA. and Cas3 positive rates were calculated as follows: positive area of immunohistochemistry/(positive area of immunohistochemistry + negative area of immunohistochemistry). All the indices (positive rates) were calculated with at least four random fields for each slide.

All results were reported as means ± SD. All statistical analyses and comparisons were done by student’s t-tests in GraphPad Prism (v6.0, GraphPad Software, Inc., San Diego, CA, USA). Statistical significance was set at *P* < 0.05.

## Results

### Cell Diversity in GC Tissue Culture

Tissue structural integrity is the fundamental basis of oncology research, as it maintains the cellular diversity in cultures (29). Fibronectin is widely found in animal tissues and tissue fluids. The most basic and important function of fibronectin is to promote the growth of cell adhesion, which is necessary for the maintenance of the tumor microenvironment and the completion of cancer cell growth. Collagen-I is the structural protein of the ECM, forming its skeleton, and anchoring and supporting tumor cells. It also provides an appropriate microenvironment for their proliferation and growth. Alpha-smooth muscle actin (α-SMA) is an isoform that is typically expressed in vascular smooth muscle cells.

**Figure 2:**
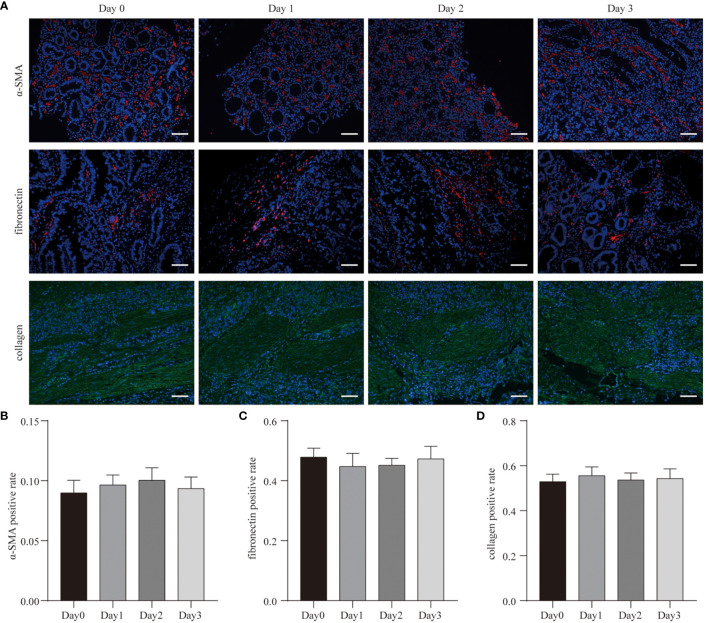
Cell diversity in GC tissue culture. **(A)** After 3 days culture, the patient-derived GC tissues (Case3) were stained with α-SMA, fibronectin, collagen, and counterstained with DAPI. Scale bars are all 100 μm. **(B–D)** Quantitative analysis of α-SMA, fibronectin, and collagen expression during the 3 days culture.

Fibronectin, collagen-I, and vascular α-SMA are tumor stromal components. Hence, we labeled them using antibodies (fibronectin, mAb; collagen-I, mAb; α-SMA, mAb) to detect changes in the tumor stroma during culture (**Figure 2A**). As shown in **Figure 2A**, after 3 days of culture, a large number of α-SMA positive cells still could be found within the tumor tissue. There were no statistical differences in α-SMA expression between the native tumor and cultured tissues (**Figure 2B**). Thus, after 3 days of culture, a large number of vascular smooth muscle cells still existed in the tumor microenvironment.

Similar to the results of α-SMA expression, there were no statistical differences in fibronectin and collagen-I expression between the native tumor and cultured tissues (**Figures 2C, D**). The morphological structures of fibronectin and collagen-I also did not change. These data suggest that in this 3D system, tumor tissues maintained structural integrity throughout the culturing periods.

### GC Cell Proliferation, Apoptosis, and Hypoxia in Culture

**Figure 3:**
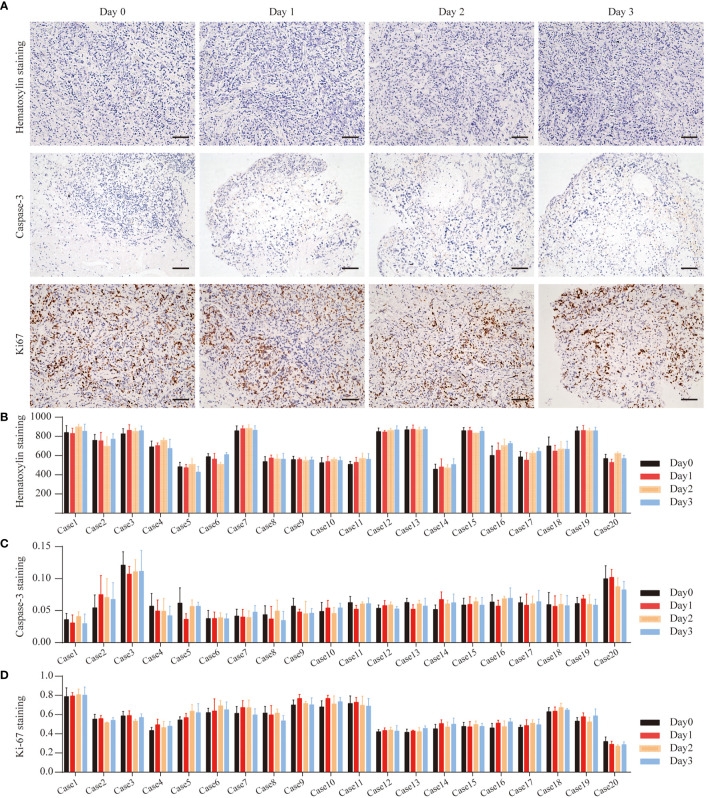
GC Cell Proliferation and Apoptosis during the culture. **(A)** Hematoxylin staining of Case13 before and after 3 days of culture. Cas3 staining of Case15 before and after 3 days of culture. Ki67 staining of Case16 before and after 3 days of culture, scale bar, 100 μm. **(B)** Quantitative analysis of hematoxylin in 20 cases of GC tissues. **(C)** Quantitative analysis of Cas3 in 20 cases of GC tissues. **(D)** Quantitative analysis of Ki67 in 20 cases of GC tissues.

To determine whether the number of cells in the tissue remained stable during culture, H&E staining was used to count the total cell numbers daily (**Figure 3A**). Based on this, we calculated the cell density (cell number/area) at different times and found no statistical changes in cell numbers prior to and after culturing (**Figure 3B**).

To determine the cell viability ratio before and after the culture (Day 0, Day1, Day2, Day3), Ki67 and Cas3 immunohistochemistry staining was used on all collected tissues to determine the cell proliferation and apoptosis indices (**Figure 3B**). **Figures 3C, D** show the overall levels of Ki67 and Cas3 expressed in five GC tissues on Day 0, Day1, Day2, and Day3. In all specimens, the majority of cells were highly proliferative prior to culturing. The proportion of apoptosis cells in almost all of the cases was less than 10%. Therefore, these tissues were considered to be suitable for culture and further drug response research.

**Figure 4:**
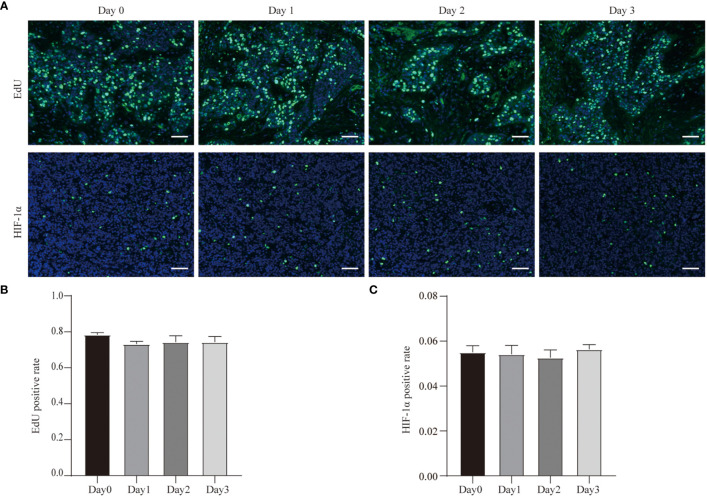
EdU incorporation and hypoxia of GC cell during the culture. **(A)** EdU incorporation of Case29 before and after 3 days of culture, scale bar, 50 μm, and HIF-1α staining of Case30 before and after 3 days of culture, scale bar, 50 μm. **(B)** Quantitative analysis of EdU in Case29. **(C)** Quantitative analysis of HIF-1α in Case30.

In order to more rigorously detect the proliferation of tumor cells in the tissue, we collected an additional sample and incubated the tissue in EdU-containing medium for 2 h before they were fixed. The results showed that the EdU levels of tumor cells in tissues didn’t change during the culture compared to the tissues which were not cultured (**Figures 4A, B**). This indicates that the tumor tissue was still in a proliferative state immediately after isolation, and its proliferation state was not affected by our 3D *ex vivo* culture system.

In addition, we also tested hypoxia in the tumor tissue during the culture process. We used HIF-1α antibodies to detect the expression of HIF-1α in tissues at different time points of the culture process. The results showed that the proportion of HIF-1α-positive cells was low in the tumor tissue that was just isolated, and the proportion of HIF-1α-positive cells did not change even after the tissues were cultured (**Figures 4A, C**). This result shows that our three-dimensional *ex vivo* culture system did not hinder the gas exchange between tumor tissue and the outside world and did not affect the tissue’s absorption of oxygen.

### Cancer Cellular and Structural Integrities Within GC Tissue

To reveal the GC cell variations in culture with time, we used cytokeratin-specific (Keratin20, a marker expressed in most of GC cells) (30, 31) and CD133 (a marker of cancer stem cells) staining. These parameters enabled us to determine the composition of GC tissues.

**Figure 5:**
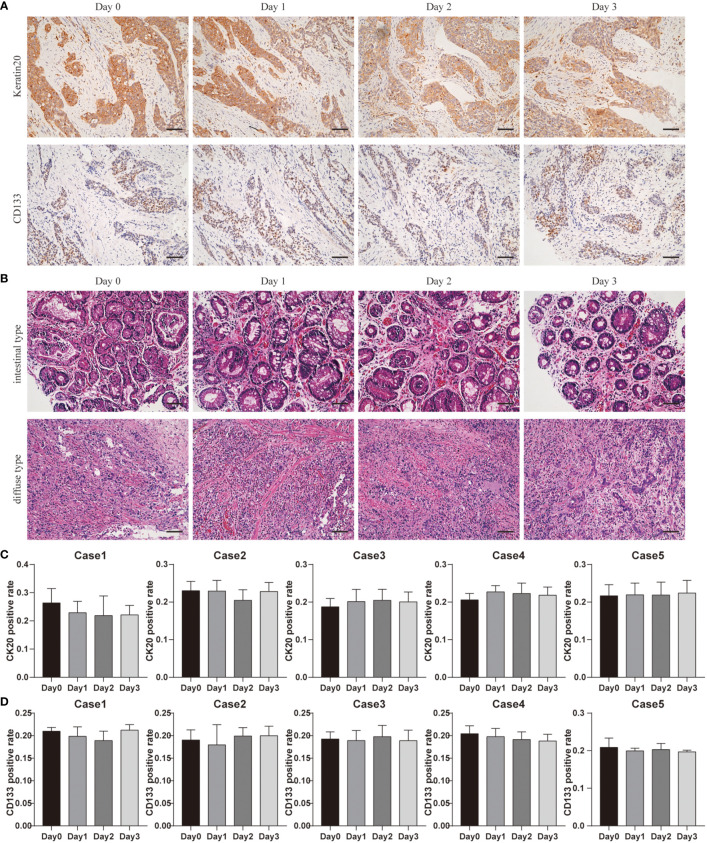
Cancer cellular and structural integrities within GC tissue. **(A)** Keratin20 staining of Case1 before and after 3 days of culture, scale bar, 100 μm. CD133 staining of Case4 before and after 3 days of culture, scale bar, 100 μm. **(B)** Case13 was intestinal type carcinoma and Case17 was diffuse gastric carcinoma, and the H&E staining at different time points were laid out. Compared with results before culture (day0), the cancer cell distributions and structure integrities were not changed a lot after 3 days of culture. All images are the same magnification, scale bar, 100 μm. **(C)** Keratin20 quantitative analysis of Case1-Case5 before and after 3 days of culture. **(D)** CD133 quantitative analysis of Case1-Case5 before and after 3 days of culture.

**Figure 5A** shows the results of Keratin20 staining in GC tissues before and after 3 days of culturing. Quantitative results demonstrated that there were no significant differences of Keratin20 in tissues isolated on Day 0 and cultured for up to 3 days (**Figure 5C**). Quantitative analyses revealed that Keratin20 expression remained unchanged throughout the whole culture period (**Figure 5C**). In most cases, Keratin20 expression was relatively stable in GC tissues during the whole culture process (**Figure 5C**), which suggested that the cancer cells can be maintained at a reasonable level during the culture period.

At the same time, we used the CD133 staining to detect GC stem cells before and after culturing (**Figure 5A**). Our quantitative results showed that the number of GC stem cells remained stable at all points of time (**Figure 5D**). These data demonstrated the reliability of our system in estimating drug response tests.

According to Lauren classification, patient-derived GC tissues can be defined as intestinal type carcinoma and diffuse gastric carcinoma; Case 13 was intestinal type carcinoma and Case 17 was diffuse gastric carcinoma. H&E staining showed that the different types of GC maintained their unique morphological structures during culturing. **Figure 5B** shows that even after 3 days in culture, the different types of GC maintained their unique morphological structures. Our classifications were confirmed by board-certified pathologists.

### Drug Responses in Culture

During the 3 culture days, we did not find significant differences in the cell distribution or structural changes between the center and edge zones of the tissue cubes. Due to the small sizes of (1–2 mm) and continuous supply of nutrients and oxygen (tissues were transplanted into 6-well plates with 1 ml culture medium with the lower segments submerged in the medium) to the tissue cubes, slight differences in nutrient diffusion and growth factor/drug permeation between the middle and peripheral parts can be ignored to some extent (32, 33). With our protocol, the ideal cell morphology and intact tissue integrity can be preserved for at least 3 days in culture, ensuring increased reliability of drug response tests.

A total of five tissues were obtained and cultured in this study. Administration of chemotherapeutics revealed that almost all specimens had decreased expression of Ki67, accompanied by varying degrees of increased Cas3 expression in culture. However, the effects of the drug were different from case to case.

**Figure 6:**
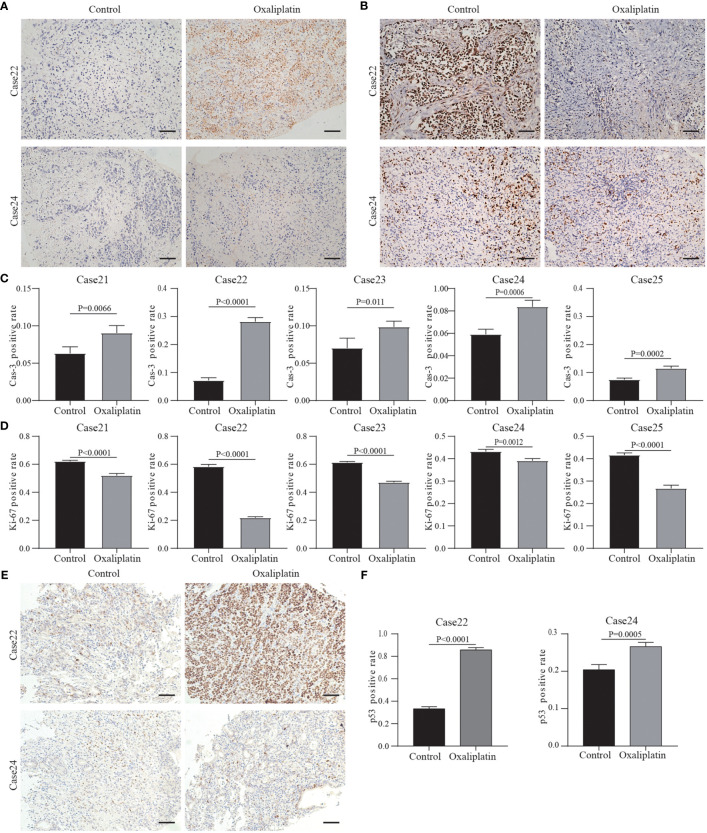
Results of drug response tests to oxaliplatin based on 3D GC tissue culture. **(A)** The tumor tissues of Case22 and Case24 were stained with Cas3 after 3 days of culture and oxaliplatin treatment. **(B)** The tumor tissues of Case22 and Case24 were stained with Ki67 after 3 days of culture and oxaliplatin treatment. All images are the same magnification, scale bar, 100 μm. **(C, D)** Quantitative analysis of Ki67 and Cas3 in tissues of Case21-25. **(E)** The tumor tissues of Case22 and Case24 were stained with p53 after 3 days of culture and oxaliplatin treatment. All images are the same magnification, scale bar, 100 μm. **(F)** Quantitative analysis of p53 in tissues of Case22 and Case24.

We selected and presented histochemical pictures of the two most resistant and sensitive tissues (Case22 and Case24). And the remaining three cases’ pictures were showed in the **Supplementary Figure 1**. Taking Case22 and Case24 as examples, **Figures 6A, B** show the drug responses of the two patients. In Case22, Ki67 expression greatly decreased following treatment with oxaliplatin, whereas Cas3 expression increased (**Figure 6C**). This suggests that oxaliplatin was a more effective chemotherapeutic agent for Case22. For Case24, Ki67 expression was maintained (only minor change) after the administration of chemotherapeutics, whereas Cas3 expression increased a little (**Figure 6D**). These results suggest that tumors from different patients have varying sensitivity to chemotherapies and that our culture system can effectively reflect this variance. These findings demonstrate the potential utility of our system in studying personalized therapies in GC. Future studies should focus on incorporating a larger patient cohort to better understand the relationships between the cell types and drug sensitivity.

Studies have shown that platinum drugs promote tumor cell apoptosis by activating P53 production (34, 35). Therefore, we tested the p53 protein levels in the tissues treated with oxaliplatin. The results showed that after treatment, the level of p53 protein in the tissues sensitive to oxaliplatin (such as Case22) was higher compared to the level of p53 protein in tissues resistant to oxaliplatin (such as Case24) (**Figures 6E, F**). The results of the remaining three cases are in the **Supplementary Figures 1C, D**. This also proved that oxaliplatin enters tumor cells to play a role in suppressing cancer.

**Figure 7:**
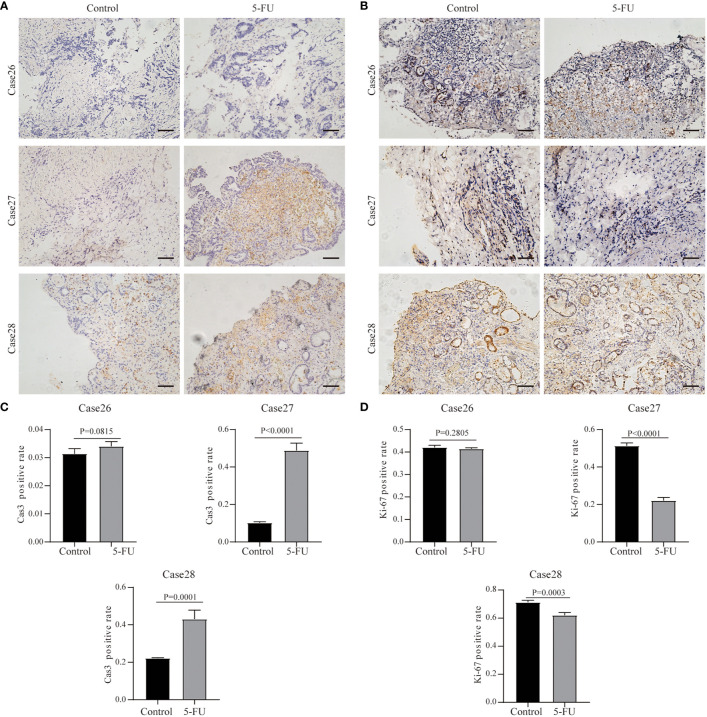
Results of drug response tests to 5-FU based on 3D GC tissue culture. **(A)** The tumor tissues of Case26, Case27, and Case28 were stained with Cas3 after 3 days of culture and 5-FU treatment. **(B)** The tumor tissues of Case26, Case27, and Case28 were stained with Ki67 after 3 days of culture and 5-FU treatment. All images are the same magnification, scale bar, 100 μm. **(C, D)** Quantitative analysis of Ki67 and Cas3 in tissues of Case26-28.

In addition, we also performed 5-FU drug sensitivity tests on three other cases. For Case26, Ki67 and Cas3 expression were maintained after the administration of chemotherapeutics, and Cas3 expression didn’t change (**Figure 7**). In Case27, the Ki67 expression greatly decreased following treatment with 5-FU, whereas Cas3 expression increased (**Figure 7**). This suggested that 5-FU was an effective chemotherapeutic agent for Case27. Case28 showed partial response to 5-FU treatment (**Figure 7**).

## Discussion

At present, GC prognosis and neoadjuvant treatments remain ineffective in the peri- and post-operative periods (36). Patient variabilities, and endogenous tumor tissue heterogeneity highlight the need for more precise and individualized treatment strategies. Especially for postoperative treatment, traditional and standardized chemotherapy regimens are not completely effective in all patients, and often contribute to severe side effects (37, 38). Even so, only 5% of patients experience a 5-year survival benefit, with most experiencing poor prognostic outcomes (5, 6).

Clinically, there is a need for the development of protocols that have high stability, shorter turnaround times, and increased accuracy in predicting treatment efficacies in individual patients. A prerequisite for a more accurate prediction is an easy-to-use laboratory protocol that can maintain cell viability, heterogeneity, and a relatively intact stroma composition, and demonstrate personalized results of drug susceptibility in a short time period following surgery (29). Currently, popular methods of investigating drug responses include the use of 2D primary cell cultures, PDX models, and organoid models. 2D primary cell culture mainly uses primary tumor cells in the culture process (39), and rarely correlates with the matrix components within primary tissue. When the system was constructed, cells other than tumor cells and extracellular matrix were excluded. This system cannot precisely reflect the subtle interactions among the cells and cell-matrices. At the same time, not all primary tumor cells can survive; only cell populations suitable for a specific culture medium can be expanded. A mouse PDX model is used for validation of novel therapies (40). However, the establishment of the model takes a long time (perhaps more than 3 months), and the success rate is low, approximately 30%. Even if a tumor is formed, the phenotype of the tumor before and after tumor formation or after passage may also change. The intrinsic time-consuming nature of PDX establishment often results in the loss of original characteristics of the primary tumor microenvironment (41). Therefore, data derived from PDX models do not reliably predict patient outcomes (42). In recent years, the construction technology of organoid models has become more advanced. However, the organoid model has problems similar to those of PDX models. The tumor formation process of the PDX model and the process of organoid establishment have a screening effect. Tumor cells that adapt to their internal environment or culture environment can proliferate, while tumor cells that cannot adapt are eliminated. This problem can be reflected in the success rate of PDX and organoid modeling.

Here we demonstrated a simple and steady *ex vivo* 3D culture protocol using patient-derived tissues and assessed the drug response to the first-line medication oxaliplatin and 5-FU. Due to the limited conditions in most hospitals, we abandoned the tissue chopper method and chose to use random dicing and grouping to build a three-dimensional culture platform. We placed the tumor tissue at the air-liquid interface for culture, so that the tumor tissue could have a sufficient oxygen supply while absorbing nutrients in the culture medium. Our data suggested that the unique specimen processing and original culture conditions accounted for tumor and patient variabilities, providing a robust system for assessing drug responses before administration. Almost all patient-derived GC tissues maintained their original viability for at least 72 h in our system. Moreover, during the process of culturing the tumor tissue, tumor cells, interstitial cells such as fibroblasts, extracellular matrix proteins such as collagen-I, and other components such as micro vessels, all maintained their original proportions, and their morphology remained unchanged. Importantly, in a small cohort, we successfully evaluated different responses to a drug regimen in different patients. Our cost- and time-effective 3D culture platform can provide personalized drug response predictions within 3 days. Our system enables reliable data acquisition and maximizes the time frame for research of resistant mechanisms. Our 3D culture platform may provide an alternative solution for assessing the outcomes of empirical medications.

However, this model also has some shortcomings that need to be clarified. The heterogeneity in tumor tissue has long been the focus of attention of scholars. In our system, heterogeneity still exists. Although we used random groupings and randomly selected fields of view for data collection and analysis to reduce the impact of heterogeneity on the results, the effect is still indelible and is reflected in the error bars in the bar graphs.

Overall, our findings demonstrated a new route for GC research. Our system is suitable for earlier prediction of drug responses and may be used for individualized treatment of GC. In addition, our new model can also be used for the development and promotion of new drugs, as well as for deeper mechanism research. Certain specific clinical studies can also be tested on this model in advance. Use of our system may also potentially decrease unnecessary side effects in patients. We believe that our 3D culture system will become a useful and effective method for future oncology research and clinical treatment.

## Conclusions

We established an innovative *ex vivo* protocol for 3D culturing patient-derived GC tissues to perform cost-effective personalized drug screening with short turnaround times. During the 3 culture days, we demonstrated the preservation of structural integrity in the tumor matrices and cell viability of all tissues. We administered different chemotherapeutic agents to cultured tissues and derived responses that were able to reliably guide treatment course for patients. Our system can be scaled for laboratory use and can be used in the determination of clinically translatable treatment plans for patients with GC.

## Data Availability Statement

The original contributions presented in the study are included in the article/**Supplementary Material**. Further inquiries can be directed to the corresponding authors.

## Ethics Statement

The studies involving human participants were reviewed and approved by the ethics committee of the Second Affiliated Hospital of Wenzhou Medical University. The patients/participants provided their written informed consent to participate in this study. Written informed consent was obtained from the individual(s) for the publication of any potentially identifiable images or data included in this article.

## Author Contributions

SC: performed the experiments, analyzed and interpreted the data, and drafted the manuscript. CC: performed the experiments and statistical analysis. YH: performed the experiments and statistical analysis. CZ: acquired the data and material support. XL: revised the manuscript and finally approved the version of the manuscript for publication. LW: revised the manuscript and finally approved the version of the manuscript for publication. XW: acquired the data and material support. XS: acquired the data and material support. XC: acquired the data and material support. WX: acquired the data and material support. HL: acquired the data and material support. XH: revised the manuscript. CL: revised the manuscript. JX: acquired the data and material support. XX: made contribution to the conception and design, analyzed and interpreted the data, supervised the study, provided the project funding, revised the manuscript and finally approved the version of the manuscript for publication. XS: made contribution to the conception and design, analyzed and interpreted the data, supervised the study, provided the project funding, revised the manuscript and finally approved the version of the manuscript for publication.

## Funding

This study was supported by grants from the National Nature Science Foundation of China (Grant Nos.: 81672707, 31670922), the Key R&D Program of Zhejiang Province (Grant No.: 2020C03029), and the Zhejiang Provincial Natural Science Foundation of China (grant No. LQ16H190003).

## Conflict of Interest

The authors declare that the research was conducted in the absence of any commercial or financial relationships that could be construed as a potential conflict of interest.

## Supplementary Material

Supplementary Figure 1 Results of drug response tests to oxaliplatin based on 3D GC tissue culture. **(A, B)** The tumor tissues of Case21, 23, and 25 were stained with Ki67 and Cas3 after 3 days of culture and oxaliplatin treatment. All images are the same magnification, scale bar, 100 μm. **(C)** The tumor tissues of Case21, Case23, and Case25 were stained with p53 after 3 days of culture and oxaliplatin treatment. All images are the same magnification, scale bar, 100 μm. **(D)** Quantitative analysis of p53 in tissues of Case21, Case23, and Case25.
