# Supplementary figures and images for: Three-Dimensional Ex Vivo Culture for Drug Responses of Patient-Derived Gastric Cancer Tissue
Source: Front Oncol. 2021 Feb 15;10:614096. doi: 10.3389/fonc.2020.614096 (PMC7917258; doi:10.3389/fonc.2020.614096)

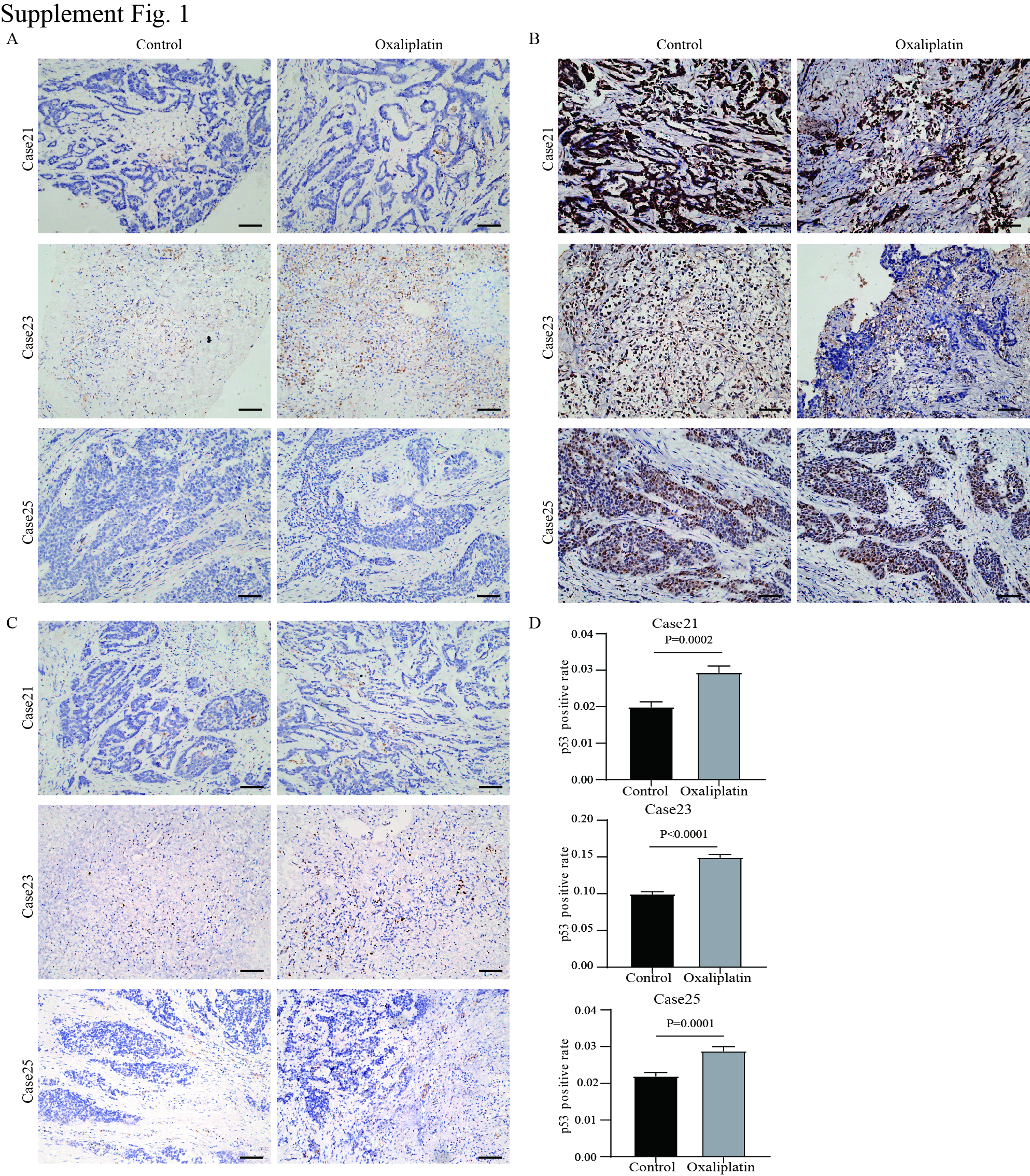

Supplement: Supplementary Figure 1 — Results of drug response tests to oxaliplatin based on 3D GC tissue culture. (A, B) The tumor tissues of Case21, 23, and 25 were stained with Ki67 and Cas3 after 3 days of culture and oxaliplatin treatment. All images are the same magnification, scale bar, 100 μm. (C) The tumor tissues of Case21, Case23, and Case25 were stained with p53 after 3 days of culture and oxaliplatin treatment. All images are the same magnification, scale bar, 100 μm. (D) Quantitative analysis of p53 in tissues of Case21, Case23, and Case25. [file Image_1.tif]
