# Supplementary material for: Three-Dimensional Ex Vivo Culture for Drug Responses of Patient-Derived Gastric Cancer Tissue
Source: Front Oncol. 2021 Feb 15;10:614096. doi: 10.3389/fonc.2020.614096 (PMC7917258; doi:10.3389/fonc.2020.614096)
Supplement: Supplementary file 2 [file Table_1.docx]

**Table S1. Summary of patients’ information and tumor characteristics.**

| **code** | **Gender** | **Age** | **Tumor location** | **Tumor size** | **Infiltration depth** | **differentiated degree** | **TNM stage** |
| --- | --- | --- | --- | --- | --- | --- | --- |
| Case1 | Male | 66 | Gastric antrum | 7cm×5.5cm×1.8cm | full-thickness | Medium-poorly differentiated | ⅢC |
| Case2 | Male | 71 | Cardia | 7cm×6cm | subserosa | Medium-poorly differentiated | ⅢB |
| Case3 | Male | 73 | Gastric angle | 2.5cm×1.8cm | submucosa | Poorly differentiated | ⅠA |
| Case4 | Male | 66 | Gastric body | 5*3.5 | Serosa | Medium differentiated | ⅢA |
| Case5 | Male | 60 | Pylorus | 6.0cm×5.0cm×1.0cm | full-thickness， | Poorly differentiated | ⅢB |
| Case6 | Female | 49 | Gastric body | 1.2cm×0.9cm×0.3cm | submucosa | Poorly differentiated | ⅠB |
| Case7 | Male | 69 | Gastric antrum | 6.5cm×4.7cm×1.5cm | Deep muscularis |  | ⅠB |
| Case8 | Male | 76 | Gastric antrum | 4cm×3cm×1.5cm | superficial muscular | Medium differentiated | ⅡB |
| Case9 | Female | 63 | Cardia | 3cm×2cm×0.5cm | submucosa | Medium differentiated | ⅠA |
| Case10 | Male | 63 | Gastric angle | 4cm×3.5cm×1.5cm | subserosa | Medium-poorly differentiated | ⅡA |
| Case11 | Male | 82 | Gastric body | 4cm×2.4cm×1.2cm | subserosa | Medium-poorly differentiated | ⅡB |
| Case12 | Male | 67 | Gastric antrum | 3cm×2cm | Deep muscularis | Poorly differentiated | ⅡB |
| Case13 | Male | 65 | Pylorus | 2cm×1.6cm | subserosa | Poorly differentiated | ⅢA |
| Case14 | Male | 51 | Gastric antrum | 6cm×6cm×1.5cm | Serosa | Poorly differentiated | ⅢA |
| Case15 | Female | 73 | Gastric body | 3.5cm×2.5cm×1.1cm | Serosa | Medium-poorly differentiated | ⅢB |
| Case16 | Female | 86 | Gastric angle | 4.4cm×2.7cm×1.3cm | submucosa | Medium differentiated | ⅠB |
| Case17 | Male | 62 | Gastric antrum | 4.5cm×3.2cm×0.6cm | superficial muscular | Medium-poorly differentiated | ⅡB |
| Case18 | Male | 50 | Pylorus | 2cm×1.5cm×1.5cm | superficial muscular | Medium-poorly differentiated | ⅡA |
| Case19 | Male | 69 | Gastric antrum | 6cm×4cm×1.2cm | full-thickness | Medium-poorly differentiated | ⅢC |
| Case20 | Female | 65 | Gastric body | 3cm×1cm×0.4cm | subserosa | Medium differentiated | ⅠA |
| Case21 | Male | 72 | Cardia | 8cm×6cm×1.5cm | subserosa | Medium-poorly differentiated | ⅡB |
| Case22 | Female | 79 | Gastric body | 6cm×4cm×1cm | Serosa | Poorly differentiated | ⅢB |
| Case23 | Male | 48 | Gastric antrum | 4cm×2.5cm×1cm | full-thickness | Poorly differentiated | ⅢC |
| Case24 | Male | 64 | Gastric angle | 3cm×2.7cm | Serosa | Poorly differentiated | ⅡB |
| Case25 | Male | 75 | Pylorus | 2.8cm×2cm | muscularis propria | Medium-poorly differentiated | ⅠB |
| Case26 | Female | 71 | Gastric body | 4cm×3cm×1cm | Serosa | Poorly differentiated | ⅢB |
| Case27 | Male | 50 | Fundus | 2.4cm×1.8cm×0.7cm | full-thickness | Poorly differentiated | ⅢA |
| Case28 | Male | 67 | Gastric angle | 1.5cm×1.5cm | subserosa | Medium-poorly differentiated | ⅡB |
| Case29 | Male | 79 | Gastric body | 4.5cm×3cm×2.4cm | Serosa | Poorly differentiated | ⅢA |
| Case30 | Female | 64 | Gastric body | 3cm×2cm | superficial muscular | Medium differentiated | ⅡB |
