# Supplementary material for: Three-Dimensional Ex Vivo Culture for Drug Responses of Patient-Derived Gastric Cancer Tissue
Source: Front Oncol. 2021 Feb 15;10:614096. doi: 10.3389/fonc.2020.614096 (PMC7917258; doi:10.3389/fonc.2020.614096)
Supplement: Supplementary file 3 [file Table_2.docx]

**Table S2. The primary and the second antibodies used in this study.**

| **Antibody** | **Target** | **Species** | **Dilution** | **Vendor** |
| --- | --- | --- | --- | --- |
| **Primary antibody** |  |  |  |  |
| Keratin20 | Cytokeratin, GC cell | rabbit anti-human | 1:800 | CST^*^, 13063 |
| Fibronectin | Fibroblast | mouse anti-human | 1:200 | Santa, sc-8422 |
| Collagen1 | collagen | rabbit anti-human | 1:100 | Abcam, ab34710 |
| α-SMA | Smooth muscle cell | rabbit anti-human | 1:320 | CST, 19245 |
| Ki67 | Proliferative and viable cell | rabbit anti-human | 1:400 | CST, 9027 |
| Cleaved caspase 3 (Cas3) | Apoptotic cell | rabbit anti-human | 1:1000 | CST, 9664 |
| CD133 | Stem cell | rabbit anti-human | 1:500 | Abcam, ab19898 |
| P53 | P53 | mouse anti-human | 1:400 | Maxim, MAB0674 |
| HIF-1α | Hypoxia | rabbit anti-human | 1:500 | Abcam, ab16066 |
| **Second antibody** |  |  |  |  |
| Alexa Fluor 568 labeled goat anti-rabbit IgG (H+L) | | | 1:200 | Invitrogen, A11011 |
| Alexa Fluor 488 labeled goat anti-rabbit IgG (H+L) | | | 1:250 | Invitrogen, A11034 |
| Alexa Fluor 555 labeled goat anti-mouse IgG1 | | | 1:250 | Invitrogen, A21127 |
| Dako REAL EnVision Detection System , Rabbit/Mouse, Working solution | | | | Dako, 20052898 |
